# Editorial: Multifactorial balance assessment, falls prevention and rehabilitation

**DOI:** 10.3389/fnagi.2025.1680310

**Published:** 2025-09-05

**Authors:** Dimitrios Kikidis, Brooke Nairn, Christos Nikitas, Marousa Pavlou, Nattawan Utoomprurkporn, Doris-Eva Bamiou

**Affiliations:** ^1^1st Department of Otorhinolaryngology, Head and Neck Surgery, National and Kapodistrian University of Athens, Hippocrateion General Hospital, Athens, Greece; ^2^The Ear Institute, Faculty of Brain Sciences, University College London, London, United Kingdom; ^3^Department of Neuro-Otology, Royal National Ear Nose Throat Hospital, University College, London Hospitals, London, United Kingdom; ^4^Chulalongkorn University Faculty of Medicine, Bangkok, Thailand

**Keywords:** balance rehabilitation, falls prevention, balance assessment, balance impairment, falls risk

Falls remain a major public health challenge, particularly among adults aged 65 and older, with one-third of individuals in this population experiencing at least one fall annually and nearly 10 % suffering recurrent events (WHO Global Report, 2007; Sui et al.). Multifactorial in origin, involving the vestibular, visual, central nervous and proprioceptive systems, falls are correlated with several comorbidities, requiring multidisciplinary assessment. This Research Topic underscores the multifactorial nature of balance impairment by combining age-related sensorimotor decline, concurrent diagnoses and the possible effect of cognitive impairment.

Epidemiological data indicate that fall rates increase with advancing age and multimorbidity, while injury-related morbidity, hospital admissions, and mortality continue to rise globally (Sui et al.; Choo et al.). The burden is both clinical and economic: substantial direct costs (e.g., hospitalizations, rehabilitation) accompany indirect sequelae (e.g., effects on quality of life, loss of independence, fear of falling, social isolation, and caregiver strain), highlighting the need for preventive strategies and interventions.

Despite evidence-based recommendations supporting balance-based fall interventions, critical gaps persist. Chief among these is the underdevelopment of comprehensive assessment tools capable of capturing cognitive, sensory, and motor domains simultaneously, which limits the individualization of therapy. There is also limited integration of dual-task and neurocognitive training into standard rehabilitation protocols, which creates a barrier to addressing real-world fall risk (Nairn et al., 2025a). Moreover, adherence remains problematic with many older adults disengaging early, with up to a 50 % dropout rate of standard falls and balance programs, suggesting a mismatch between intervention design and patient-centered factors (Pavlou et al., 2013; Simek et al., 2012). Finally, the full value of indirect benefits including reduced caregiver burden and enhanced self-confidence, remains largely uncaptured in current evaluations, impeding policy buy-in.

Current trends in clinical practice reflect a gradual shift from single-domain interventions to multifactorial programs tailored to individual needs (Elrod and Wong). Established exercise protocols such as the Otago protocol (Wang and Kim) are being supplemented with dual-task and cognitive components to address fall risk more holistically and personally (Nairn et al., 2025a; Liston et al., 2014). Emerging studies highlight the integration of telerehabilitation and augmented reality, which offer scalable and remotely deliverable care models, especially vital in underserved regions (Gulline et al., 2025). However, real-world adoption remains inconsistent: many clinics continue to focus on generic exercise modules without adjusting for cognitive load, comorbidities, or patient lifestyle, indicating a need for stronger implementation frameworks.

Looking ahead, future directions must harness emerging technologies and methodological refinements to bridge existing gaps while meeting patient needs. Wearable biosensors and markerless motion analysis systems show promise for dynamic, real-time risk stratification. Advanced computational models integrating environment, physiology, and patient behavior may enable predictive analytics and personalized intervention pathways. Virtual reality solutions have recently become commercially available; however, their role is limited by default since they cannot facilitate walking exercises which are essential for the reestablishment of mobility.

Intensive research has been conducted using cutting-edge augmented reality technology within the context of three EU-funded projects (HOLOBALANCE, SMART BEAR, and the current TELEREHAB DSS). These projects project a holographic physiotherapist avatar in real-space, providing exercise programme guidance. These platforms also capture movements via body motion tracking sensors for providing real-time feedback on patients exercise performance and corrections. Preliminary work investigating these platforms' acceptability, feasibility and effectiveness has already been completed. The results have shown promise, in terms of cost-effectiveness, patient acceptability and usability among patients with MCI (Bovornratanaraks et al., 2024; Utoomprurkporn et al., 2023) and stroke (Nairn et al., 2025) in addition to preliminary feasibility using IMUs, pressure-sensitive wellness mats and the eHealth literacy application (Georgas et al.). Market analysis has also proven, that these telerehabilitation solutions fill many existing gaps and make rehabilitation more accessible and engaging, with TeleRehab DSS standing out as superior in terms of AI-decision support, objective data collection and real-time feedback (Nairn et al., 2025b).

Other pressing issues highlighted by Research Topic include the need for standardized outcome metrics. Currently, measures vary widely making study comparison difficult. A unified core outcome set would advance research coherence and meta-analytic capacity. Workforce development also requires attention: implementing multifactorial programs demands training physiotherapists in cognitive assessment and dual-task facilitation, in addition to the dedicated skills needed for balance physiotherapy which are not currently part of the relevant curricula, and creating multidisciplinary teams with clearly delineated roles while avoiding siloed delivery. Finally, equity considerations are paramount: older adults with neurological comorbidities or from socioeconomically disadvantaged backgrounds face the greatest fall risk, but are often excluded from trials. Future research must emphasize inclusion and access to ensure that interventions reach those most in need.

In summary, this Research Topic provides a timely and rigorous examination of multifactorial balance assessment and rehabilitation. By highlighting epidemiology, identifying intervention gaps, outlining current practices and trends, and charting future opportunities, this Research Topic offers a roadmap for translating science into sustainable, equitable fall prevention strategies.
